# Access to hospice and palliative care for people with a migration background: a qualitative study on challenges and recommendations in end-of-life care

**DOI:** 10.1186/s12904-026-02116-x

**Published:** 2026-05-07

**Authors:** Sabahat Ölcer, Annika Albert, Maximiliane Jansky, Friedemann Nauck, Christian Banse

**Affiliations:** 1 Department of Palliative Medicine, University Medical Center Göttingen, University of Göttingen, Von-Siebold-Straße 3, Göttingen, 37075 Germany; 2 Department of Human-Centered Technology Development, Institute of Computer Science, Ruhr West University of Applied Sciences, Campus Bottrop, Bottrop, Germany

**Keywords:** Hospice and palliative care, Patients with a migration background, Social determinants of health, Health policies, Healthcare providers, Qualitative research

## Abstract

**Background:**

Systemic inequalities shape healthcare access, disproportionately affecting people with a migration background in Germany. Empirical studies on this group in hospice and palliative care are quite limited and highlight a gap in understanding the challenges and potential solutions.

**Objectives:**

To explore, from the perspectives of healthcare providers, the challenges faced by patients with a migration background, their families, and healthcare providers in hospice and palliative care. The aim was to improve accessibility, reduce barriers, and ensure that hospice and palliative care services are accessible and inclusive to them.

**Methods:**

A qualitative study was conducted employing directed content analysis of healthcare providers’ responses to open-ended questions on end-of-life care. The analysis was guided by a system-facing social determinants of health framework, emphasising systemic and structural factors influencing care services. Healthcare providers recruited from hospice and palliative care facilities across Germany (*n* = 332).

**Results:**

Language barriers, cultural insensitivity, and systemic inequalities were identified by healthcare providers as major obstacles hindering access to hospice and palliative care for patients with a migration background. Healthcare providers emphasised the need for tailored support services, community-based networks and other innovative solutions to improve accessibility and outcomes. Moreover, they highlighted that the emotional burden on all actors involved in hospice and palliative care is exacerbated by language barriers, cultural misconceptions, and the structural complexity of end-of-life care.

**Conclusion:**

Prioritising equitable and culturally sensitive care as part of broader support services and innovative solutions can enable broad participation in hospice and palliative care, including patients with a migration background and their families, based on mutual trust. Further research is needed to overcome logistical and financial challenges and facilitate the implementation of actionable recommendations within hospice and palliative care services.

**Supplementary Information:**

The online version contains supplementary material available at 10.1186/s12904-026-02116-x.

## Background

As immigration increases and countries and communities grow more culturally and linguistically diverse, it becomes crucial to address systemic inequalities and to ensure equal opportunities for all individuals, particularly those with a migration background [1, 2]. Studies suggest that there is a cumulative disadvantage for people with a migration background, including unequal access to healthcare services [3–6]. Vulnerable circumstances, such as family separation due to immigration, unfavourable living environment, difficult working conditions and experiences of exclusion and discrimination, can negatively impact both health status and integration into the healthcare system [7, 8]. Furthermore, language and communication difficulties, different cultural backgrounds and religious beliefs, lack of knowledge about available healthcare services, and insufficient consideration of immigration-specific circumstances by the healthcare system represent barriers to accessing healthcare services [9, 10]. These factors result in this population group utilising healthcare services less and being underrepresented in healthcare facilities [7, 9, 11].

Barriers to accessing hospice and palliative care (HPC) are even more noticeable than in other healthcare areas [11, 12]. HPC is characterised by the care of patients with life-limiting illnesses and a strong focus on quality of life, symptom management, and supportive care. It typically involves close collaboration between different professional groups, such as physicians, nurses, and social workers [13]. Family members are also often closely involved in care processes and decision-making. In these settings, care often goes beyond standard therapeutic interventions as patients confront the deep vulnerabilities associated with terminal illness and its consequences and the realities of death and dying [13]. The care process becomes more complicated for people with a migration background due to language barriers, cultural differences, and limited familiarity with the healthcare system. These factors make it harder to ensure equitable and patient-centred end-of-life care [12, 14].

Achieving health equity in access to and delivery of high quality services requires more research focused on the unique needs of this population group and the development of innovative approaches to overcome existing barriers [15]. The social determinants of health (SDoH) [16, 17] framework provides a comprehensive lens to examine inequalities in healthcare access, particularly for patients with a migration background (PwM). This framework helps to explain how broader social, economic, and environmental factors shape the opportunities and constraints that PwM face in accessing care. It also allows a more holistic understanding of how structural and systemic conditions influence individual health experiences and outcomes in HPC. In this study, we applied a system-facing perspective [18], using the SDoH framework to interpret how healthcare providers observe these systemic factors shaping the experiences of PwM in everyday care.

Empirical studies on this topic specific to Germany are quite limited. There is a significant gap in the literature to fully understand the challenges in this context and to address the potential solutions [7, 11, 19]. Our study aimed to explore the challenges faced by PwM, their families, and healthcare providers across HPC services from the perspective of healthcare providers. We also examined what policies and practical measures could help promote the involvement of PwM in HPC and improve access to support services. In this context, the study identified structural, cultural, and communication barriers that affect care delivery and highlighted ways to make palliative care more inclusive and culturally sensitive.

## Methods

### Study design

We drew on concepts from the SDoH framework as outlined by the World Health Organization (WHO) [16] and discussed in Duquaine-Watson [17], including social and community context, economic stability, health and healthcare access, neighbourhood and built environment, and education access and quality. These concepts guided the development of our coding categories, explicitly linking the analysis to the SDoH model and highlighting how systemic factors influence healthcare access and outcomes for PwM and their families. The framework guided the analysis of the data collected from HPC facilities through an online survey that also included open-ended questions. It helped to explore the challenges faced by actors, including healthcare providers, PwM and their family members, from the perspective of healthcare providers. It also provided recommendations to reduce systemic inequalities in healthcare access and outcomes and enabled a system-level perspective on how structural conditions shape these barriers. A directed qualitative content analysis approach [20, 21] was used for data analysis. We applied SDoH concepts to develop our coding and better understand access barriers in HPC.

### Recruitment and participants

Staff from palliative care units, inpatient hospice services, specialised palliative home care teams, volunteer hospice services, palliative care physicians, and palliative advisory teams in hospitals in Germany were invited to participate. Targeting healthcare providers in HPC facilities across Germany was achieved through a non-probability voluntary response sampling. The German Association for Palliative Medicine provided a table with the email addresses of most HPC institutions in Germany to facilitate the recruitment process. This information was derived from the comprehensive online directory ‘*Wegweiser Hospiz- und Palliativversorgung* (Guide to Hospice and Palliative Care)’ [22], which lists the majority of facilities nationwide. The survey link was sent to these institutional email addresses with the request to forward it to a healthcare provider working at the facility who was eligible to participate.

Table 1 provides an overview of the participants’ characteristics, the types of facilities they represented, and the countries of origin of the last treated PwM. The participant group was diverse, with the majority being female and mainly aged between 51 and 60 years. Most professions included coordinators, doctors, and nurses, which showed a broad range of healthcare roles. In terms of care settings, outpatient hospice services, specialised palliative home care, and palliative care units were the most common. The last patients treated came from various countries, with Turkey, Russia, and Syria being the top three countries of origin.

**Table 1:** Participants’ characteristics, type of facilities, and countries of origin of the last treated PwM

Categories	Characteristics	*n*	%	Categories	Characteristics	*n*	%
Gender	Male	77	23.2	Type of facility (Multiple answers possible)	Palliative care units	60	18.1
	Female	254	76.5		Inpatient hospice services	49	14.8
	Diverse	1	0.3		Specialised palliative home care services	96	28.9
Age	up to 20 years	0	0		Nursing services	4	1.2
	21–30 years	10	3		Volunteer hospice services	147	44.3
	31–40 years	35	10.5		Palliative care physicians	17	5.1
	41–50 years	75	22.6		Palliative medicine consult services	22	6.6
	51–60 years	166	50		Palliative hospital advisory teams	21	6.3
	61–70 years	42	12.7		Other	7	2.1
	over 70 years	4	1.2	Countries of origin of the last treated patients	Turkey	70	21.1
Profession	Physicians	82	24.7		Russia	40	12
	Nurses	71	21.4		Syria	30	9
	Psychologists	6	1.8		Poland	16	4.8
	Social workers	28	8.4		Albania	11	3.3
	Spiritual workers	2	0.6		Italy	11	3.3
	Volunteers	4	1.2		Greece	10	3
	Coordinators	120	36.1		Romania	10	3
	Others	19	5.7		Afghanistan	8	2.4
					Iraq	8	2.4
					Iran	8	2.4
					Ukraine	7	2.1
					Other countries of origin or country of origin unknown	103	31

### Data collection

Our online survey including open-ended questions was adapted from previously published instruments. The core content was based on the questionnaire developed by Jansky et al. [23], who surveyed specialised palliative care providers on challenges in end-of-life care for people with a Turkish or Arab migration background in Lower Saxony. Items were drawn from prior literature. Moreover, items were partially adapted from the representative survey on health and hospital policy by the German Hospital Institute [24] and supplemented with study-specific modifications (see Supplementary File 1) [9]. The questionnaire prioritised open-ended questions, which allowed respondents to freely express their opinions. It included questions aimed at eliciting the experiences and thoughts of healthcare providers caring for PwM on issues, such as possible access barriers, recommendations for action, case descriptions of the most recently cared for patient as well as identified problem areas and solutions in care, specific migration-related offers in facilities, strategies related to the care of this patient group, and cooperation with other facilities.

All institutions registered as providing services for adult patients in the voluntary and self-report *Wegweiser Hospiz- und Palliativversorgung* were invited to participate in the online survey (*n* = 2711). These included palliative care units (*n* = 332), inpatient hospice services (*n* = 236), specialised palliative home care services (SAPV; *n* = 288), outpatient hospice services (*n* = 1123), palliative care physicians (*n* = 652), palliative care consult services (*n* = 13), and hospital-based palliative care services (*n* = 67). The *open-source software ‘formR’* (version v0.17.19), developed by Arslan and Tata [25, 26], was employed to create an online questionnaire (AA, CB, MJ, and FN). The online survey was sent to respondents via email on November 27, 2019, and closed to them on February 1, 2020 (AA). Due to invalid email addresses, questionnaire invitations were successfully sent to 2413 facilities. After excluding incomplete responses, a total of 332 completed questionnaires were included in the analysis.

### Data analysis

We analysed the responses to the open-ended questions in the questionnaire for this study. The initial coding was conducted by AA and SÖ with the help of MAXQDA software (version 2020) [27]. The directed qualitative content analysis approach [20, 21] was applied to the text analysis, guided by the concepts from the SDoH framework [16, 17]. First, responses to open-ended questions were read several times and examined through the lens of the framework to establish their relevance to predefined theoretical concepts. Key ideas, recurring patterns, and initial impressions that could inform the subsequent coding were identified during the familiarisation stage. Second, using predefined coding categories derived from the SDoH, responses were systematically coded (SÖ). For example, responses discussing cultural and language barriers, family involvement, and encouraging community and patient engagement were categorised under social and community contexts. Issues related to emotional burden, resource limitations, and increased demand for support were assigned to the concept of health and healthcare access (see Supplementary File 2). To ensure consistency and reliability, all authors compared and discussed coding decisions to resolve discrepancies and achieve consensus. After coding, the data were collaboratively synthesised within each domain, which revealed challenges identified by healthcare providers and practical recommendations to improve palliative care for PwM.

### Ethical considerations

Respondents participated in the online survey after being informed about the purpose of the study and providing their consent. The Institutional Review Board of the University Medical Center Göttingen approved the study protocol and final questionnaire (application no. 20/10/19, dated 11.05.2019), with no ethical or legal concerns. All study procedures were conducted in accordance with the World Health Organization’s Declaration of Helsinki. The abbreviation HP1/2/…/332 (Healthcare Provider) was used to identify respondents, ensuring their anonymity.

## Results

Healthcare providers identified challenges and actionable recommendations for three key actors: PwM, their families, and the healthcare providers themselves (Table 2). We identified five themes and their categories in line with the SDoH.

**Table 2:** Healthcare provider perspectives on system-facing challenges and policy recommendations for PwM, family members, and themselves

				Actors		
Social determinants of health categories	Subcategories	Challenges	Policy recommendations	PwM	Family members	Healthcare providers
Social and community context	Language access and communication	Language barriers Access to interpreters	Language support services	x	x	
			Multilingual counselling hotlines		x	
	Building cultural competence and sensitivity	Cultural sensitivity Lack of awareness	Religious support services	x		
			Cultural competency training		x	x
			Community-based support networks		x	x
			Developing comprehensive guidelines			x
	Social support and family involvement	Family involvement	Support for family caregivers	x		
			Organised family meetings		x	
			Advance care planning		x	
			Family as decision-makers		x	x
	Building trust in healthcare system	Mistrust toward healthcare providers	Promoting trust through community involvement	x	x	
			Self-help activities	x		
			Combating healthcare discrimination	x		
Economic stability	Financial support and accessibility	Financial concerns	Simplified financial guidance	x		
			Providing financial literacy training		x	x
Health and healthcare access	Psychosocial support for actors	Emotional burden Increased demand for support	Culturally relevant sensitive psychosocial support services	x		
			Peer-support groups	x		
			Family counselling		x	
			Psychosocial support	x	x	x
	Enhancing language support services	Language barriers Resource limitations	Access to interpretation services			x
			Training healthcare providers to work with interpreters			x
			Multilingual communication aids	x	x	x
Neighbourhood and built environment	Legal considerations	Immigration status Bureaucratic barriers	Safe access to care regardless of legal status	x		
			Legal aid services	x	x	x
	Housing and safe living environments	Housing instability	Housing assistance	x		
Education access and quality	Training and health literacy	Low health literacy	Health literacy programs on palliative care	x	x	
			Patient navigators in palliative care	x		

### Social and community context

*Language access and communication*. Healthcare providers identified language barriers as a significant obstacle for PwM in HPC services. These barriers often cause misconceptions about treatment and care instructions and hinder emotional support. One healthcare provider explained, *“[there was] a lack of understanding about the progress of the disease*” (HP166). Despite PwM frequently relying on family members for interpretation, their support was more likely to lead to confusion and miscommunication. As healthcare providers stated:

*“Husband speaks little German; interpreter is not reliably available.”* (HP3)

*“The husband translated*,* but didn’t translate properly; he only said what he thought his wife should hear.”* (HP84)

The findings revealed the critical need for professional interpreters and translators as free language services, particularly when access is often limited. One healthcare provider noted, “*with conversations by phone and directly with the translator*” (HP306). Moreover, healthcare providers emphasised overcoming language barriers by “*building trust through independent interpreters*” (HP92). They further highlighted the importance of 24-hour availability of multilingual counselling hotlines to ensure timely and effective communication. As another healthcare provider suggested, *“24-hour availability of contact persons …”* (HP214).

*Building cultural competence and sensitivity*. Healthcare providers observed that discrepancies between the cultural expectations of PwM and the approach of the palliative care team can lead to mistrust or reluctance to follow advice. Providers’ lack of awareness of cultural practices and religious beliefs could result in family members using pressure on the patient regarding treatment decisions. Healthcare providers explained:

*“Was only allowed to be cared for by a male nurse.”* (HP118)

*“Problem of defining the therapeutic goal. For ethical and religious reasons*,* it was difficult to convey the decision not to use resuscitation measures and further invasive diagnostics/therapy.”* (HP219)

*“There was no understanding of how to deal with the relatives and their death-related cultural practices.”* (HP242)

*“Muslim citizens are concerned about attending Christian-influenced institutions.”* (HP79)

The actionable recommendations from healthcare providers were religious support services for PwM and cultural competency training. One healthcare provider emphasised, “*further training in dealing with other religions and cultures*” (HP24). They also suggested community-based support networks for family members and healthcare providers and comprehensive guidelines for providers. As another healthcare provider noted, “*hospice volunteer qualification course deals with religious and spiritual guidelines*,* rituals*,* etc. in a themed evening*,* including the care of the deceased in different cultures*” (HP29). Some healthcare providers further recommended closer collaboration with faith communities and clearer procedures for action:

*“Recommendations for action drawn up by the team on how to deal with people with a migration background and their relatives in order to understand the ‘behaviour’ and to respond sensitively to it*,* but also to show boundaries (for staff*,* to protect fellow patients)”* (HP155)

*“Closely involve the Islamic community (as an example).”* (HP9)

*“Procedures for action have been established for people of the Muslim faith*,* both for treatment and after death.”* (HP155)

*Social support and family involvement*. Healthcare providers indicated difficulties in navigating family dynamics, especially when PwM relied on family members for guidance and support, or when families were unable to come to terms with death and dying. One provider described how “*the children were often overwhelmed with half-true information about their mother’s health condition*” (HP44). Moreover, frequent visits by family members and relatives also caused problems both in the workflow of healthcare providers and in the well-being of other patients:

*“The patient was cared for by many relatives*,* which was a problem for some of the nursing staff. The neighbouring ward also had issues with the large number of relatives.”* (HP242)

Family involvement is crucial in care services for PwM, as families often play a central role in support and decision-making: “*Regular conversations with all household members (4 generations ranging from 3 years to 78 years old)*” (HP176). Providers recommended adopting structured approaches to enhance outcomes, such as family meetings, advance care planning, and fostering active family involvement in care decisions while addressing barriers and providing adequate resources for effective engagement. As other healthcare providers noted:

*“External family/children counselling in advance.”* (HP20)

*“Many conversations with all parties involved [and] constant availability of the palliative care physician.”* (HP232)

*“Many intensive conversations*,* relatives were constantly involved in the care*,* the consistent attitude of the entire team.”* (HP311)

*Building trust in healthcare system*. Another subcategory of the social and community context of the SDoH domain was building trust in the healthcare system. Respondents stressed that mistrust of healthcare systems or providers may be more evident among PwM and their families, mainly if they have previously experienced a negative emotional situation in their homeland or if healthcare systems are not inclusive or respectful of their cultural values. Healthcare providers stated:

*“Distrust towards the providers*,* questioning whether everything is being done in terms of medical possibilities.”* (HP6)

*“In the extreme situation*,* it was almost impossible to build trust. The hospice volunteers were seen as strangers*,* and due to the experiences in the home country*,* scepticism remained.”* (HP191)

Building trust and equitable care for PwM involved community outreach programs to raise awareness of HPC, engage immigrant organisations, and foster self-help groups: “*Offering information events directly at immigrant organisations and communities*” (HP238). According to some healthcare providers, collaborating with community leaders and ensuring culturally sensitive care could help combat discrimination and misconceptions and encourage them and their families to seek care when needed:

*“More self-help activities by immigrants*,* e.g. through self-help groups.”* (HP90)

*“Language problems (rare Kurdish dialect)*,* but these were solved by volunteers from hospice work*,* as we have volunteers from various nations and some of them speak more than three languages each. Cultural diversity and misunderstandings between nurses*,* doctors and the patient were also resolved by volunteers.”* (HP69)

*“Respectful*,* polite interaction.”* (HP242)

### Economic stability

*Financial support and accessibility*. Economic challenges were often mentioned by healthcare providers as significant barriers to accessing HPC for PwM and their families. Limited financial resources frequently restricted patients’ options and mobility, especially in rural areas, and hindered access to necessary services such as nursing or palliative care. Some healthcare providers noted:

*“Her time options were very limited because the financial situation was tight*,* and she had to work during that time.”* (HP184)

*“Financial worries (limited mobility because she lived in a rural area and had no car available).”* (HP81)

*“The person was unable to afford a nursing service.”* (HP234)

Economic challenges also highlight the necessity for healthcare providers to receive training on how to discuss financial concerns with PwM in a culturally sensitive manner. They also pointed out that multilingual financial counselling could help them and their families understand healthcare costs, insurance coverage, and available public benefits. One healthcare provider described the complexity of such processes as involving “*permanent phone calls with the social welfare office/health insurance company*,* high personal commitment*” (HP33), while another reported that “*the patient was transferred to the residential hospice within 14 days*,* with costs approved by the social welfare provider/county administration in an individual case decision*” (HP42).

### Health and healthcare access

*Psychosocial support.* Healthcare providers reported feeling emotionally strained when caring for PwM. The combination of language barriers, cultural misunderstandings, and the complex nature of HPC made communication and emotional support challenging. One healthcare provider shared that relatives “*pushed for further oncological therapy*” and perceived *“refusal … as devaluation and unequal* treatment” (HP119). In addition, healthcare providers stated that many PwM feel anxious, isolated, and distressed during end-of-life care due to language and cultural differences, which affected their psychosocial health. These experiences further demonstrated that both family members and healthcare providers require additional psychosocial support in the care process. As healthcare providers emphasised:

*“Despite many conversations with the hospice service and hospice volunteers*,* the relatives were unable to accept the course of the illness or participate in palliative care.”* (HP69)

*“High emotionality*,* expectations regarding therapy options*,* and difficulty coping with the illness.”* (HP83)

Healthcare providers recommended culturally sensitive psychosocial support services (e.g. therapy in the patient’s mother tongue and in the context of their cultural background and religious beliefs) and peer-support groups (e.g. exchanging experiences and advice with patients from similar cultural backgrounds) for PwM, family counselling (e.g. establishing counselling hotlines) for their families, and psychosocial support for all involved:

*“Further training on dealing with illness*,* death*,* dying and mourning in other cultures.”* (HP306)

*“The Romanian priest here often came to translate and provide psychosocial and pastoral care.”* (HP115)

*“Conversations with caring relatives to support them in dealing with illness in the family.”* (HP176)

*Enhancing language support services*. Healthcare providers frequently highlighted the challenges posed by language barriers in delivering effective care to PwM and their families: “*Due to language barriers*,* there is insufficient information about the relevant offers*” (HP1). One healthcare provider explained that while “*communication with the patient in German was unproblematic*,” problems arose when “*an interpreter was necessary*,* leading to difficulties in synchronising information and agreements*” (HP206). Similarly, another healthcare provider described how limited language skills made it hard to provide emotional support, noting that “*the grief and emotional pain could only be inadequately absorbed by the nursing staff*” (HP115).

These barriers hindered clear communication and limited access to available support services. Healthcare providers emphasised the need for sustainable solutions, including the use of certified medical interpreters, professional translation services, and multilingual materials. As one healthcare provider noted, “*regular contact with interpreters and language training*” were key to improving care quality (HP206). Another healthcare provider highlighted the importance of “*early involvement of interpreters*” and providing “*information material in native languages*” (HP256). Some also suggested drawing on “*employees with their own immigration biography and language skills*” (HP69) to bridge cultural and linguistic gaps.

### Neighbourhood and built environment

*Legal considerations and safe living environments*. PwM and their families face bureaucratic and legal obstacles that prevent access to necessary care and support services. Challenges such as residency permits for family members and navigating asylum processes on healthcare benefits further exacerbated their vulnerability during end-of-life care. As one healthcare provider noted, a patient’s wife “*faced considerable hurdles from the asylum authorities*” (HP130) when trying to visit her dying husband. The other pointed out that restrictions in refugee law meant that essential services such as nursing care were unavailable for months:

*“The refugee law provides for medical services but not nursing care; this entitlement only applies after 8 months – this means that people in palliative situations fall through the social safety net.”* (HP42)

Living in inadequate housing or not having access to safe environments conducive to end-of-life care were other challenges. As healthcare providers have stated, some PwM received end-of-life care in settings that were far from ideal:

*“Long distance between the palliative care unit and the family’s place of residence.”* (HP15)

*“Patient was accommodated in a refugee centre*,* no water in the room*,* toilet*,* shower and kitchen in the basement without relatives or friends*,* only spoke Russian foreseeably.”* (HP42)

To address these challenges, healthcare providers suggested solutions such as ensuring safe access to care regardless of legal status and enacting new legislation to improve access. They also recommended providing legal aid and housing support in HPC to help PwM and their families, as healthcare providers emphasised:

*“The embassy in Berlin was contacted*,* and the passport was issued. The parents in India were contacted through a doctor in our region originally from India. An organisation providing assistance for the return journey was contacted. Medical and organisational preparation and execution of the return journey to India. This was an absolutely gigantic project !!!”* (HP296)

*“Placement of wife in a flat close to [X] (a university hospital in Germany)”* (HP233)

*“Development of a care structure in the accommodation centres for asylum seekers with our own staff as a model project.”* (HP130)

### Education access and quality

*Training and health literacy*. Healthcare providers pointed out the challenges in meeting the informational and care management needs of PwM and their families in the German healthcare system. Unfamiliarity with healthcare provider roles, for example why “*the palliative care physician is not an emergency doctor*” (HP205), and uncertainty about where to seek help often led to confusion: “*Complete lack of knowledge regarding the German healthcare system (e.g. What are emergency rooms for? What is the role of the general practitioner? )*” (HP205). Language barriers further intensified these challenges, contributing to misunderstandings about key concepts such as “*hospice*” and “*palliative care*” (HP8). In some cases, relatives expressed a need for further explanations and repeatedly questioned clinical decisions, with one healthcare provider noting:

*“Relatives (each of the 12 children) have an extremely high need for information and aggressively demand it and doubt the competence of the treating physicians.”* (HP189)

To improve HPC access for PwM, healthcare providers recommended funding health literacy programs tailored to different languages and literacy levels. Multilingual patient navigators would enhance understanding of care options, healthcare rights, and decision-making processes while increasing awareness of care in this group. One healthcare provider described how their team even developed a “*schedule … with addresses*,* tips*,* etc. for people with a Turkish migration background*” (HP155), while another noted the value of “*various external and internal training courses on the topic*” (HP212).

## Discussion

Our study provides a comprehensive perspective on the challenges encountered by various actors in HPC, framed by the SDoH. It presents system-facing solutions identified by healthcare providers. These solutions foster the involvement of PwM and their families in care while addressing related challenges.

Our findings highlight the critical need for professional medical interpreters [28, 29], multilingual resources [11], and culturally sensitive training for healthcare providers [30–34]. Language barriers affect communication as well as the overall delivery of care [35, 36]. However, 24/7 access to interpreters in multiple languages supports equal opportunities, but it also presents major logistical and financial challenges and adds a substantial burden on healthcare costs [37, 38]. In this system-facing context [18], the potential use of culturally sensitive speech-based systems, such as tools that support communication by recognising different languages and cultural expressions, may offer innovative solutions [39–41]. Studies on their use in HPC may provide cost-effective and accessible solutions to mitigate language barriers while maintaining cultural sensitivity and improving the quality of care.

Our results extend existing knowledge by linking cultural competence gaps to mistrust and suboptimal care [11, 42–45]. They also point to targeted interventions such as religious support services, community-based networks, and collaboration with immigrant organisations to build trust and promote equal care [46, 47]. These findings emphasise the value of sharing resources across institutions and community-based immigrant organisations rather than placing the responsibility solely on the healthcare system and related facilities. This approach helps alleviate institutional pressures and encourages the strengths of community networks to provide culturally relevant and accessible support. Moreover, increasing awareness of HPC within the community may facilitate greater use of care services within this population.

Our study deepens the discussion on systemic inequalities, such as economic and structural barriers in care. It highlights how financial instability, legal challenges, and inadequate housing intersect, factors that have been less explored in the context of PwM in Germany [48, 49]. It also addresses the emotional burden experienced by PwM, their families, and healthcare providers in HPC. Emotional burden is often exacerbated by a lack of culturally sensitive care and language barriers [9, 50, 51]. Our findings suggest that culturally appropriate psychosocial support services [51] and peer support networks [52] may help reduce psychological health needs, while tailored health literacy programs [12, 14, 15] and patient navigators [12, 50] may improve HPC access and outcomes for this group. However, the specific barriers to implementing these interventions within care services require further investigation. However, system-facing barriers to implementing these interventions within care services require further investigation.

### Strengths and limitations

A key strength of this study is that it provides system-facing insights into the challenges encountered in diverse societies and immigration contexts, such as Germany. It highlights the multiple challenges affecting PwM, their families, and healthcare providers in palliative care and offers actionable recommendations for improving care delivery. While our study focuses on the German context, our findings are also relevant to other countries experiencing immigration-related challenges in HPC.

Although the study is comprehensive, it may not be commonly applicable to all PwM and HPC settings in Germany. In addition, the response rate to the survey was relatively low. The invitation was distributed to a large number of heterogeneous institutions listed in the *Wegweiser Hospiz- und Palliativeversorgung*, and in many cases the email was sent to general institutional addresses rather than to specific professionals, which may have reduced the likelihood of participation. The data were collected in 2019–2020. Despite societal changes since then, including the COVID-19 pandemic and the war in Ukraine, the main findings regarding the challenges faced by healthcare providers in palliative care for PwM remain relevant. Moreover, many of the identified challenges still persist in practice, highlighting the ongoing relevance of these findings. However, certain contextual aspects, such as service delivery models, access restrictions, and institutional workload, may have changed. The responses to open-ended questions provided a broad perspective on the issues, but written responses limited interpretability, and further exploration was not possible. In addition, although participants represented different professional groups, we did not conduct a systematic comparison of responses by profession, as the study aimed to capture a broad system-level perspective rather than profession-specific differences. Importantly, our study reflects only the perspectives of healthcare providers and does not include the direct voices of PwM or their families. This presents a methodological limitation. Future research should incorporate patients’ and families’ own experiences to complement and deepen the system-facing insights presented here.

## Conclusion

Integrating AI-based support systems, such as speech-based systems with cultural sensitivity, into HPC services can help reduce communication and language barriers and ease logistical and financial pressures. Strengthening collaboration between healthcare systems and community-based organisations can also support trust-building and improve access to culturally sensitive care. Future research on system-facing approaches is needed to develop sustainable ways of implementing these interventions. Including the perspectives of PwM and their families will also be essential for improving HPC outcomes for diverse populations. This could be achieved by conducting qualitative studies, such as in-depth interviews or focus group discussions. Collaboration with community-based organisations and cultural mediators could further facilitate access, enhance trust, and ensure that interventions are culturally appropriate and relevant.

## Supplementary Information

Supplementary Material 1.

Supplementary Material 2.

## Data Availability

The datasets generated and/or analysed during the current study are not publicly available due ethical restrictions but are available from the corresponding author on reasonable request.

## Abbreviations

PwM: Patients with a migration background
HPC: Hospice and palliative care
SDoH: Social Determinants of Health
MAXQDA: Max Weber Qualitative Data Analysis
HP: Healthcare provider
