# Supplementary material for: Access to hospice and palliative care for people with a migration background: a qualitative study on challenges and recommendations in end-of-life care
Source: BMC Palliat Care. 2026 May 7;25:131. doi: 10.1186/s12904-026-02116-x (PMC13154880; doi:10.1186/s12904-026-02116-x)
Supplement: Supplementary file 1 — Supplementary Material 1. [file 12904_2026_2116_MOESM1_ESM.docx]

**Supplementary File 1. Online questionnaire on hospice and palliative care for people with a migration background in German** [1, 2, 3]^[[1]](#footnote-1)^

**1.** **Information about the institution/organisation**

**a)** What type of institution do you work in? (Multiple answers possible)

- Palliative care unit
- Inpatient hospice service
- Specialised palliative home care service
- Nursing service
- Volunteer hospice service
- Palliative care physician in private practice
- Palliative medicine consultation service
- Palliative hospital advisory team
- Other
- Please specify other institutions here:…………………………………………………
- To which of the institutions you have mentioned would you like to refer in your following responses?..............................................................................................

**b)** In which federal state (Bundesland) is your institution located?

- Baden-Württemberg
- Bavaria
- Berlin
- Brandenburg
- Bremen
- Hamburg
- Hesse
- Mecklenburg-Vorpommern
- Lower Saxony
- North Rhine-Westphalia
- Rhineland-Palatinate
- Saarland
- Saxony
- Saxony-Anhalt
- Schleswig-Holstein
- Thuringia

**c)** Where is your institution located?

- Major city with >500,000 inhabitants
- Large city with >100,000 inhabitants
- Small town with <100,000 inhabitants
- Rural region

**d)** Which professional groups work in your institution? (Multiple answers possible)

- Physicians
- Nurses
- Psychologists
- Social workers
- Spiritual workers
- Volunteers
- Coordinators
- Others

**2.** **Patients with migration background in your institution**

- The Federal Statistical Offices [4] defines individuals with a migration background as follows:

“*A person has a migration background if they themselves, or at least one parent, did not acquire German citizenship by birth.*”

​

This definition encompasses both the absence of citizenship and any citizenship other than German as indicating a migration background.

**a)** Assessment of migration status in the patient history

To what extent are the following information routinely inquired about during the patient history assessment?

Patient’s nationality Yes/No

Patient’s country of birth Yes/No

Parents’ country of birth Yes/No

Patient’s religious/spiritual affiliation Yes/No

Patient’s native language Yes/No

Patient’s language proficiency Yes/No

Need for interpretation services in the course of care Yes/No

- The Federal Statistical Office [4] defines individuals with a migration background as follows:

“*A person has a migration background if they themselves, or at least one parent, did not acquire German citizenship by birth.*”

**Reminder:** Please refer your responses to the institution you previously selected (page 4 of the questionnaire).

**b)** How many patients with a migration background received hospice/palliative care in your institution in 2018? ........................................................................................

This information is:

- Estimated
- Exact

**c)** How many patients in total received hospice/palliative care in your institution in 2018? ........................................................................................

This information is:

- Estimated
- Exact
- The Federal Statistical Office [4] defines individuals with a migration background as follows:

“*A person has a migration background if they themselves, or at least one parent, did not acquire German citizenship by birth.*”

**d)** From your perspective, are there access barriers to hospice and palliative care for this patient group in your care region? Yes/No

Please briefly name the access barriers here: ..................................................................

**3.** **Case description of a patient with migration background**

- The Federal Statistical Office [4] defines individuals with a migration background as follows:

“*A person has a migration background if they themselves, or at least one parent, did not acquire German citizenship by birth.*”

This definition encompasses both the absence of citizenship and any citizenship other than German as indicating a migration background.

In the following part of the questionnaire, we would like to ask you specifically about the care of a patient with a migration background. Please think now of the last patient with a migration background who was cared for in your institution.

- I am thinking of a patient.
- No patient with a migration background has been cared for in my institution so far.
- Patient demographics and background

**a)** Patient’s age at the time of care

- Up to 20 years
- 21 to 40 years
- 41 to 60 years
- 61 to 80 years
- Over 80 years

**b)** Patient’s gender

- Male
- Female
- Divers

**c)** Type of migration background

- Patient migrated themselves
- At least one parent migrated
- Unknown

Country/Countries of origin: ........................................................................

Patient’s nationality:

- German
- Non-German

**d)** Main diagnosis: ........................................................................

**e)** Reason for admission to hospice/palliative care: ...........................................................

**f)** Were there problems...

- ...in the care of the patient? Yes/No

Which problems were these? .......................................................................................

Were the problems resolved? Yes/No

How were the problems resolved? ...............................................................................

- ...with relatives and/or associates? Yes/No

Which problems were these? .......................................................................................

Were the problems resolved? Yes/No

How were the problems resolved? ...............................................................................

- ...with other caregivers? Yes/No

Which problems were these? .......................................................................................

Were the problems resolved? Yes/No

How were the problems resolved? ..............................................................................

**g)** Were there difficulties in communication with the patient, relatives, and/or acquaintances? Yes/No

- Was a professional interpreter involved for translation in this case? Yes/No
- How satisfied were you with the involvement of an interpreter?

|  |  |  |  |  |
| --- | --- | --- | --- | --- |

Very satisfied Not satisfied at all

- Was staff with the corresponding language skills involved for translation in this case?

Yes/No

- How satisfied were you with the involvement of staff with the corresponding language skills?

|  |  |  |  |  |
| --- | --- | --- | --- | --- |

Very satisfied Not satisfied at all

- Were relatives, friends, or acquaintances of the patient involved for translation in this case? Yes/No
- How satisfied were you with the involvement of relatives, friends, or acquaintances of the patient?

|  |  |  |  |  |
| --- | --- | --- | --- | --- |

Very satisfied Not satisfied at all

**h)** Did ethical problems arise that led to conflicts? Yes/No

Please describe the situation in more detail: ..........................................................................

Could a satisfactory solution be reached for all parties involved? Yes/No

**i)** How satisfied were you retrospectively with the patient’s care?

|  |  |  |  |  |
| --- | --- | --- | --- | --- |

Very satisfied Not satisfied at all

**j)** Do you have any further comments regarding the patient’s care? Yes/No

**4. Care of people with migration background in your institution**

**Reminder:** Please refer your responses to the institution you previously selected (page 4 of the questionnaire).

**a)** Are there already specific action recommendations for the care of people with migration background in your institution (issued by management/leadership)?

- Yes, available
- Specifically in planning
- Planned for the future
- Not available
- I don’t know
- Does the implementation of the action recommendations get regularly reviewed?
- Yes
- No
- I don’t know
- How helpful do you find the action recommendations?

|  |  |  |  |  |
| --- | --- | --- | --- | --- |

Not helpful at all Fully helpful

**b)** Are there migration-specific services for the care of people with migration background in your institution?

**Organisational services**

- Special menu selection according to religious requirements
- Yes, available
- Specifically in planning
- Planned for the future
- Not available
- I don’t know
- Is this service regularly used?
- Yes
- No
- I don’t know
- How helpful do you find this service?

|  |  |  |  |  |
| --- | --- | --- | --- | --- |

Not helpful at all Fully helpful

- Offer for consideration of migration-specific aspects in accommodation (e.g., prayer and farewell rooms)
- Yes, available
- Specifically in planning
- Planned for the future
- Not available
- I don’t know
- Offer for consideration of migration-specific aspects in handling deceased persons
- Yes, available
- Specifically in planning
- Planned for the future
- Not available
- I don’t know
- Training/continuing education offers on migration-specific topics for staff
- Yes, available
- Specifically in planning
- Planned for the future
- Not available
- I don’t know
- List of regional contacts for major religions
- Yes, available
- Specifically in planning
- Planned for the future
- Not available
- I don’t know
- Information on cultural and religious specificities for staff
- Yes, available
- Specifically in planning
- Planned for the future
- Not available
- I don’t know

**Communication**

- List of professional interpreters for translation with patients who have limited/no German language skills
- Yes, available
- Specifically in planning
- Planned for the future
- Not available
- I don’t know
- List of multilingual staff for translation with patients who have limited/no German language skills
- Yes, available
- Specifically in planning
- Planned for the future
- Not available
- I don’t know
- Communication aids/pictures/models
- Yes, available
- Specifically in planning
- Planned for the future
- Not available
- I don’t know
- Use of multilingual forms
- Yes, available
- Specifically in planning
- Planned for the future
- Not available
- I don’t know
- Is this service regularly used?
- Yes
- No
- I don’t know
- How helpful do you find this service?

|  |  |  |  |  |
| --- | --- | --- | --- | --- |

Not helpful at all Fully helpful

- Which forms are available in other languages? (Multiple answers possible)
- Consent declaration
- Information/notice sheets for stay
- Information materials on various diseases
- Others
- Please specify these other forms here: ....................................................................
- Add another service from your institution? ..................................... ………………….

**c)** To what extent are the following persons involved as translators in your institution for patients with limited/no German language skills?

- Medical staff with relevant language skills
- Never
- Occasionally
- Often
- Always
- Nursing staff with relevant language skills
- Never
- Occasionally
- Often
- Always
- Other staff with relevant language skills
- Never
- Occasionally
- Often
- Always
- Patient’s relatives/friends/acquaintances
- Never
- Occasionally
- Often
- Always

- Professional interpreters
- Never
- Occasionally
- Often
- Always

- Professional video interpreters
- Never
- Occasionally
- Often
- Always

- Roommates/other patients with relevant language skills
- Never
- Occasionally
- Often
- Always

**d)** Have you developed your own strategies in the hospice/palliative care team for the care of people with migration background?

- Yes
- No
- I don’t know

Which strategies are these?

Strategy 1: ............................................................................................................................

Add another strategy? .........................................................................................................

**e)** Does your institution/service regularly collaborate with other institutions or persons? Yes/No

- With which of the following institutions or persons does your institution/service regularly collaborate? (Multiple answers possible)
- Religious communities
- Universities
- Migration services of welfare associations
- Counselling and treatment facilities specialised in people with migration background
- Migrant (self-help) organisations
- Cultural mediators/mediators/health guides
- Others
- Please specify these additional cooperation partners here: ......................................

**f)** How important do you consider the sensitisation for migration-specific health care?

- Currently
- Not important at all
- Rather unimportant
- Rather important
- Very important
- Long-term
- Not important at all
- Rather unimportant
- Rather important
- Very important

**5. Employees with migration background in your institution**

The Federal Statistical Office [4] defines individuals with a migration background as follows:

“*A person has a migration background if they themselves, or at least one parent, did not acquire German citizenship by birth*.”

**a)** Are there employees with migration background working in your institution? Yes/No

- What type of migration background do the employees have? (Multiple answers possible)
- Employee migrated themselves
- At least one parent migrated
- Unknown
- What profession do the employees with migration background have? (Multiple answers possible)
- Nursing staff
- Psychologists
- Social workers
- Spiritual workers
- Physiotherapists
- Volunteers
- Coordinators
- Others
- Please specify other professions here: ................................................................
- Are they preferentially deployed in the care of people with migration background? Yes/No

**b)** Have employees in your institution acquired special competencies in the care of patients with migration background (e.g., further training, language courses...)? Yes/No

- Which competencies are these? Please name them briefly: ................................

**6. Demographic data**

**a)** What is your gender?

- Male
- Female
- Divers

**b)** What is your age?

- Up to 20 years
- 21 to 30 years
- 31 to 40 years
- 41 to 50 years
- 51 to 60 years
- 61 to 70 years
- Over 70 years

**c)** Which professional group do you belong to?

- Physicians
- Nursing staff
- Psychologists
- Social workers
- Spiritual workers
- Physiotherapists
- Volunteers
- Coordinators
- I belong to another professional group
- Which professional group do you belong to? ...............................................................

**7. Conclusion**

**a)** I wish for the following support in the care of people with migration background:

..............................................................................................................................

**b)** I have the following additional comments regarding the questionnaire:

................................................................................................................................

**References:**

1. Jansky M, Owusu-Boakye S and Nauck F. Palliative Versorgung von Menschen mit türkischem oder arabischem Migrationshintergrund in Niedersachsen. Bundesgesundheitsblatt-Gesundheitsforschung-Gesundheitsschutz. 2016;60(1):45-54. <https://doi.org/10.1007/s00103-016-2473-x>
2. Blum K, Löffert S, Offermanns M, et al. Krankenhaus Barometer - Umfrage. Deutsches Krankenhausinstitut, Düsseldorf, <https://www.dki.de/sites/default/files/2019-01/2017_11_kh_barometer_final.pdf>. (2017, accessed 28 October 2021).
3. Albert A. Hospiz-und Palliativversorgung von Menschen mit Migrationshintergrund [dissertation]. Göttingen: Universitätsmedizin Göttingen; 2024. <https://ediss.uni-goettingen.de/handle/11858/15438>.
4. Statistisches Bundesamt, Bevölkerung und Erwerbstätigkeit. Bevölkerung mit Migrationshintergrund – Ergebnisse des Mikrozensus 2017. <https://www.destatis.de/DE/Themen/Gesellschaft-Umwelt/Bevoelkerung/Migration-Integration/Publikationen/Downloads-Migration/migrationshintergrund-2010220177004.pdf?__blob=publicationFile>. (2017, accessed 28 October 2021).

1. For the German original survey, see Albert A. Hospiz- und Palliativversorgung von Menschen mit Migrationshintergrund [Doktora tezi]. Göttingen: Universitätsmedizin Göttingen; 2024:156-182. <https://ediss.uni-goettingen.de/handle/11858/15438> [↑](#footnote-ref-1)
