# Supplementary material for: Access to hospice and palliative care for people with a migration background: a qualitative study on challenges and recommendations in end-of-life care
Source: BMC Palliat Care. 2026 May 7;25:131. doi: 10.1186/s12904-026-02116-x (PMC13154880; doi:10.1186/s12904-026-02116-x)
Supplement: Supplementary file 2 — Supplementary Material 2. [file 12904_2026_2116_MOESM2_ESM.docx]

**Supplementary File 2. Coding tree illustrating the development of categories, sub-categories, and example codes**

- Social determinants of health categories
- Subcategories
- Challenges
- Policy recommendations
- Codes are presented as illustrative examples and do not represent the full dataset. This coding tree provides an overview of how qualitative data were organised into categories and subcategories during the analysis.
- Social and community context
- Language access and communication
- Language barriers
- Language barriers (patients & relatives)
- No German skills
- Misunderstandings
- Limited language proficiency
- Access to interpreters
- Lack of professional interpreters
- Limited interpreter availability
- Need for interpreter support
- Lack of professional interpreter use in complex communication situations
- Interpreter not reliably available
- Language support services
- Multilingual bereavement counselling
- Language-skilled hospice mentors
- Language courses for staff
- Staff with language skills
- Multilingual counselling hotlines
- Counselling hotlines
- Hotline for specific advice
- Multilingual counselling structures
- Building cultural competence and sensitivity
- Cultural sensitivity
- Filtered information (“only what patient should hear”)
- Gender-related care preferences (e.g. refusal of female staff)
- Cultural norms around truth-telling
- Religious needs
- Emotional expression styles
- Lack of awareness and understanding
- Misunderstanding of palliative care
- Lack of insight into illness and dying
- Unrealistic expectations about recovery
- Religious support services
- Freelance Muslim counsellor
- Contact with religious dignitaries
- Contact with religious groups
- Religious support by Imam
- Cultural competency training
- Advanced training on religious/cultural issues
- Increasing team knowledge on cultural practices
- Awareness raising among staff
- Religion-specific training
- Community-based support networks
- Networking with migrant organisations
- Cooperation with communities
- Networking with communities
- Developing comprehensive guidelines
- Creation of standards
- Procedures for specific religious groups
- Structured care approaches
- Standardised documents
- Guidelines
- Handbooks
- Social support and family involvement
- Family involvement
- No conversations with patient alone
- Strong family control over care decisions
- Family conflicts affecting care
- Limited direct communication with PwM
- Family determining communication and care processes
- Support for family caregivers
- Supporting family in care process
- Early involvement of family
- Space for family
- Continuous collaboration with relatives
- Organised family meetings
- Regular communication with family
- Structured family communication
- Enquiries and structured exchange
- Regular family meetings
- Advance care planning
- Early clarification processes
- Early clarification of wishes
- First-visit planning and decision-making
- Family as decision-makers
- Family involvement in care decisions
- Family co-determining care pathways
- Building trust in healthcare system
- Mistrust toward healthcare providers
- Limited cooperation with providers due to mistrust
- Mistrust of staff
- Fear-based mistrust (e.g. deportation concerns)
- Suspicion about withholding treatment
- Perception of “not doing enough”
- Promoting trust through community involvement
- Close cooperation with communities
- Building relationships with patient environments
- Self-help activities
- Self-help groups
- Community-led initiatives
- Migrant-led initiatives
- Combating healthcare discrimination
- Acceptance of diversity
- Avoiding stereotyping
- Equal care regardless of religion
- Respectful interaction
- Respectful treatment
- Economic stability
- Financial support and accessibility
- Financial concerns
- Unclear cost coverage
- Insurance-related financial issues
- Financial strain within families
- Ongoing financial insecurity
- Concerns about feasibility of care wishes (e.g. returning home country, home care)
- Simplified financial guidance
- Counselling centres for financial and social law problems
- Offering guidance/support on financial matters
- Health and healthcare access
- Psychosocial support for actors
- Emotional burden
- Difficulty coping with dying
- High emotional strain at end of life
- Fear, anxiety, despair
- Emotional overload of family
- Grief and inability to cope
- Difficulty coping with dying
- Extreme reactions (e.g. suicide attempt, collapse)
- Increased demand for support
- 24-hour care needs due to symptoms
- High care demands from families
- High need for emotional, communicative, and decision-making support
- Complex family counselling needs
- Culturally relevant sensitive psychosocial support services
- Culturally adapted counselling approaches
- Tailored psychosocial approaches
- Peer-support groups
- Peer-support groups within migrant communities
- Community peer networks
- Family counselling
- Intensive communication with families
- Addressing family needs and expectations
- Psychosocial support
- Bereavement counselling services
- Access to psychological support
- Enhancing language support services
- Language barriers
- Difficulty explaining prognosis
- No discussion of therapy
- Inability to discuss therapy or prognosis
- Resource limitations
- Lack of multilingual nursing services
- Constraints in providing culturally adapted care
- Access to interpretation services
- Interpreting services
- 24h availability
- Easy/uncomplicated access
- Online interpreting systems
- Increased availability of interpreters
- Training healthcare providers to work with interpreters
- Structured integration of interpreters in communication processes
- Structured interpreter coordination training
- Training in working with medically qualified interpreter
- Multilingual communication aids
- Information material in native languages
- Multilingual forms
- Multilingual documents
- Translated materials
- Culturally adapted flyers
- Neighbourhood and built environment
- Legal considerations
- Immigration status
- Unclear residence status
- Asylum uncertainty
- Transnational family separation impacting care
- Family members abroad
- Transnational care challenges
- Bureaucratic barriers
- Problems with authorities
- Legal/authorisation ambiguities
- Legal/social law restrictions (e.g. refugee law limiting services)
- Safe access to care regardless of legal status
- Social law coordination in asylum procedures
- Cooperation with migration services
- Public authorities (indirect structural access support)
- Legal aid services
- Asylum social counselling
- Legal counselling
- Social law support
- Housing and safe living environments
- Housing instability
- No adequate place for care after discharge
- Living in refugee accommodation
- Unsuitable home environments for care
- Threat of homelessness
- Housing assistance
- Co-supervision in asylum accommodations
- Support in difficult living environments
- Education access and quality
- Training and health literacy
- Low health literacy
- Difficulty understanding illness, prognosis, and care needs
- Difficulty understanding medical information
- Problems navigating care
- Misunderstanding of medical concepts (e.g. palliative sedation)
- Health literacy programs on palliative care
- Awareness campaigns
- Providing information to patients/families
- Training about hospice/palliative care
- Patient navigators in palliative care
- Contact point
- Dedicated contact persons and case managers
- Community representatives as intermediaries
